# Associations between the depressive symptoms, subjective cognitive function, and presenteeism of Japanese adult workers: a cross-sectional survey study

**DOI:** 10.1186/s13030-020-00183-x

**Published:** 2020-05-04

**Authors:** Kuniyoshi Toyoshima, Takeshi Inoue, Akiyoshi Shimura, Jiro Masuya, Masahiko Ichiki, Yota Fujimura, Ichiro Kusumi

**Affiliations:** 1 Department of Psychiatry, Graduate School of Medicine, Hokkaido University, Kita 15, Nishi 7, Sapporo, 060-8638 Japan; 2 Department of Psychiatry, Tokyo Medical University, Shinjuku-ku, Tokyo, 160-0023 Japan

**Keywords:** Cognitive complaints in bipolar disorder rating assessment, Cognitive dysfunction, Depression, Employment, Occupational functioning, Patient health questionnaire 9, Work limitations questionnaire 8

## Abstract

**Background:**

Presenteeism has attracted much attention in the research into mental health. However, how cognitive complaints and depressive symptoms affect presenteeism remains unknown. Therefore, this study examined the correlation between subjective cognitive impairment, depressive symptoms, and work limitations.

**Methods:**

We collected data from 477 adult workers in Japan. We evaluated subjective cognitive function using the Cognitive Complaints in Bipolar Disorder Rating Assessment (COBRA), depressive symptoms with the Patient Health Questionnaire 9 (PHQ-9), and work limitations with the Work Limitations Questionnaire 8 (WLQ-8). The relations between depressive symptoms, cognitive complaints, and work limitations were examined using Spearman’s rank correlations and multiple regression analysis. It was hypothesized that cognitive complaints would mediate the effects of depressive symptoms on work productivity loss, which was tested using path analysis.

**Results:**

The results indicated that cognitive complaints were significantly correlated with work limitations and depressive symptoms. Multiple regression analysis, using the WLQ-8 productivity loss score as the dependent variable, revealed that COBRA and PHQ-9 scores were significant predictors of work productivity loss. We performed path analysis using PHQ-9, COBRA, and WLQ-8 productivity loss scores and created a path diagram, which revealed that the direct effects of both depressive symptoms and cognitive dysfunction on work productivity loss were statistically significant. Moreover, depressive symptoms indirectly affected work productivity loss through subjective cognitive impairment. There was no significant interaction effect between depressive symptoms and cognitive complaints.

**Conclusions:**

Our results suggest that work limitations may be predicted by not only depressive symptoms but also cognitive complaints. Moreover, subjective cognitive impairment may mediate the effect of depressive symptoms on presenteeism among adult workers.

## Background

In recent years, the relation between work productivity and depression has attracted much attention [1]. Depression has been found to be highly correlated with workplace limitations in interpersonal/psychological functioning, time management, and overall work productivity [2]. However, workers with depression or in a depressive state continue to go to work, owing to factors such as financial stress and organizational policies; this could also be referred to as presenteeism, a phenomenon where an individual attends work despite being unwell [3, 4]. Absenteeism, conversely, is the state of being absent from work because of health-related impairments. Presenteeism leads to work productivity loss due to health issues [5] and is more associated with depression than absenteeism, which indicates a tendency for depressive individuals to work while sick instead of taking time off [6]. Employer-based insurance costs the employer two or three times more for medical services than direct medical care costs, including insurance premiums and pharmacy costs [7]. It has been reported that the average company has an annual loss of $617 per employee due to the compensation formula and $649 due to managing depression-related workplace disruption, compared to $316 due to conflict resolution [1].

Work-focused interventions for employed adults with depression have been developed and shown to be superior to general care in reducing depressive symptoms, absenteeism, and presenteeism [8]. In one study, the presumed cost of productivity averaged $6041.70 per subject every year [8]. Furthermore, a work-focused intervention involving cognitive-behavioral therapy techniques was effective even at a four-month follow up [9].

Presenteeism is a key outcome of cognitive dysfunction in depression [10]. Among South Korean patients with major depressive disorder, regardless of its severity, those who had more severe perceived cognitive decline reported worse work productivity [11]. However, to our knowledge, the correlation between cognitive complaints and the presenteeism of adult workers has not been studied.

The purpose of this research was to investigate the correlation between cognitive complaints and the presenteeism of adult workers from a community sample. We hypothesized that subjective cognitive impairment would be correlated with the presenteeism of adult workers and that presenteeism could be explained by depressive symptoms and subjective cognitive impairment.

## Methods

### Participants

Adults aged 20 years and over were recruited as participants via convenience sampling between April 2017 and April 2018 at Tokyo Medical University, Tokyo, Japan. In the study, inclusion criteria were as follows: (a) aged at least 20 years old; (b) not having serious physical illness; (c) no organic brain damage; and (d) having the capability to provide agreement to participate in this research. We excluded those who were not currently employed and those who did not complete the assessments. This research was approved by the Local Ethics Committee of Tokyo Medical University (Ethics Approval Number: 2016–144) in accordance with the Declaration of Helsinki. A total of 597 individuals provided written informed consent after receiving an explanation about the study, of whom 119 did not complete their questionnaires and one was not currently employed; hence, the final sample comprised 477 participants.

### Assessments

Clinical and sociodemographic data were collected from the 477 participants. Additionally, established instruments were used to evaluate their subjective cognitive function, depressive symptoms, and presenteeism.

#### Subjective cognitive function

The Cognitive Complaints in Bipolar Disorder Rating Assessment (COBRA) is a 16-item self-reported assessment for measuring subjective cognitive impairment [12]. All of the items are assessed using a four-point scale, and the total score is calculated by summing the scores across all items. The highest score is 48, with lower scores indicating lower levels of perceived neurocognitive impairment. The COBRA was first created by the Bipolar Disorder Program at the Hospital Clinic of Barcelona [12]; the Spanish version of the COBRA has been translated into Japanese [13]. The International Society for Bipolar Disorders Targeting Cognition Task Force has recommended COBRA for use as a screening tool for subjective cognitive impairment [14].

Previous studies have shown that the COBRA can be used to evaluate the subjective neuro-cognition of bipolar patients, as well as patients with depression and healthy individuals [12, 15, 16]. Further, the association between COBRA scores and life quality assessments was significant in remitted bipolar patients [17]. In Japan, COBRA has been used to evaluate subjective cognitive function in the general adult population [18]. The Japanese version of the COBRA has been validated and used in research [13, 17].

#### Presenteeism

The Japanese Work Limitations Questionnaire 8 (WLQ-8) is a shorter version of the Japanese WLQ-25. It rates health-related working disability across the four dimensions of physical demands, time management, mental-interpersonal demands, and output demands [2, 19]. The recall period for responses is the prior 2 weeks, with five choices being used for scoring: *always* (100%), *most of the time*, *some of the time* (~ 50%), *rarely*, and *never* (0%; 2,19). These subscale scores enabled us to calculate loss of work productivity using the WLQ index score, which is the weighted sum of each WLQ subscale score [19]. The WLQ work productivity loss score indicates the estimated percentage of presenteeism, and higher scores indicate higher levels of presenteeism [20]. Validation studies of the Japanese version of the WLQ-8 have been performed in which its validity and reliability have been demonstrated [21–23].

#### Depressive symptoms

The Patient Health Questionnaire-9 (PHQ-9) was developed as a self-administered scale for screening and evaluating the severity of depression [24]. The validity of the Japanese version has been confirmed [25], and its summary score (ranging from 0 to 27 points) was utilized for analysis in this study. Higher scores indicate higher levels of depressive symptoms.

### Statistical analyses

A Kolmogorov-Smirnov test was performed to check whether the COBRA, PHQ-9, and WLQ-8 scores had a normal distribution; none were found to be normally distributed (*p* < .001). Spearman’s correlation was thus used to evaluate relations among the scores on the COBRA, PHQ-9, and WLQ, as well as clinical parameters. For basic comparisons according to subjective cognitive function, participants were divided into two groups according to a cutoff COBRA score (COBRA ≤14 and COBRA > 14), and non-parametric analyses (Mann-Whitney U test) were used. Multiple regression analysis was conducted with the WLQ-8 productivity loss score as the dependent variable and COBRA and PHQ-9 scores as independent variables. Before evaluating the interaction effect, centering was performed on the mean scores. Subsequently, we performed a path analysis to examine the mediational role of cognitive complaints on the relation between depression and the loss of work productivity of adult workers. All statistical analyses were conducted with IBM SPSS Statistics 23.0 (Armonk, NY: IBM Corp.), and path analysis was performed using STATA 16 (College Station, TX: StataCorp LLC); statistical significance was set at *p* < .05.

## Results

Sociodemographic and clinical data are summarized in Table 1. The data of 477 individuals were included in this research. The average age was 41.11 (± 11.99) years, 211 (44.3%) were men, and 303 (64.1%) were married. The mean years of education was 14.72 (± 1.80). A total of 53 (11.1%) had a psychiatric history, 19 (4.0%) were currently in psychiatric treatment, and 50 (11.5%) had a family history of psychiatric treatment. A total of 314 (65.8%) participants drank alcohol and 96 (20.1%) smoked. The mean PHQ-9 score was 4.23 (± 4.30) and the mean WLQ-8 productivity loss score was 0.042 (± 0.042). The mean COBRA score was 8.45 (± 6.53), which was lower, that is, better, than the scores reported in previous studies with Japanese euthymic bipolar patients. For example, a COBRA score of 13.63 (± 7.95) was found for euthymic bipolar patients [13]. The percentage of workers who scored higher than the criterion value on the COBRA (> 14) was 18.3.

**Table 1:** Clinical and sociodemographic characteristics

Participant characteristics	Mean (*SD*) *n* (%)
Age, years, mean (*SD*) (*n* = 477)	41.11 (11.99)
Male sex, *n* (%) (*n* = 476)	211 (44.3)
Married, *n* (%) (*n* = 473)	303 (64.1)
Years of education, mean (*SD*) (*n* = 477)	14.72 (1.80)
Psychiatric history, *n* (%) (*n =* 477)	53 (11.1)
Current psychiatric treatment, *n* (%) (*n =* 472)	19 (4.0)
Family history of psychiatric treatment, *n* (%) (*n =* 434)	50 (11.5)
Drinking, *n* (%) (*n =* 477)	314 (65.8)
Smoking, *n* (%) (*n =* 477)	96 (20.1)
PHQ-9, mean (*SD*) (*n =* 477)	4.23 (4.30)
WLQ-8 Time management, mean (*SD*) (*n =* 477)	16.06 (20.52)
WLQ-8 Physical activities, mean (*SD*) (*n =* 477)	15.64 (25.44)
WLQ-8 Mental-interpersonal activities, mean (*SD*) (*n =* 477)	15.02 (17.87)
WLQ-8 Output activities, mean (*SD*) (*n =* 477)	14.81 (18.88)
WLQ-8 Index, mean (*SD*) (*n =* 477)	0.044 (0.045)
WLQ-8 Productivity loss, mean (*SD*) (*n =* 477)	0.042 (0.042)
COBRA total score, mean (*SD*) (*n =* 476)	8.45 (6.53)
COBRA total score > 14, *n* (%) (*n =* 476)	87 (18.3)

*PHQ-9* Patient Health Questionnaire-9; *WLQ-8* Work Limitations Questionnaire; *COBRA* Cognitive Complaints in Bipolar Disorder Rating Assessment

**Table 2:** Spearman rank correlation coefficients among COBRA, PHQ-9, and WLQ-8 scores (*N* = 476)

	COBRA	PHQ-9
PHQ-9	.407**	–
WLQ-8 Time management	.399**	.358**
WLQ-8 Physical activities	.222**	.174**
WLQ-8 Mental-interpersonal activities	.431**	.382**
WLQ-8 Output activities	.409**	.307**
WLQ-8 Index	.470**	.399**
WLQ-8 Productivity loss	.470**	.399**

*COBRA* Cognitive Complaints in Bipolar Disorder Rating Assessment; *PHQ-9* Patient Health Questionnaire-9; *WLQ-8* Work Limitations Questionnaire; **p < 0.01 (two-sided)

### Subjective cognitive function and work limitations

**Fig. 1:**
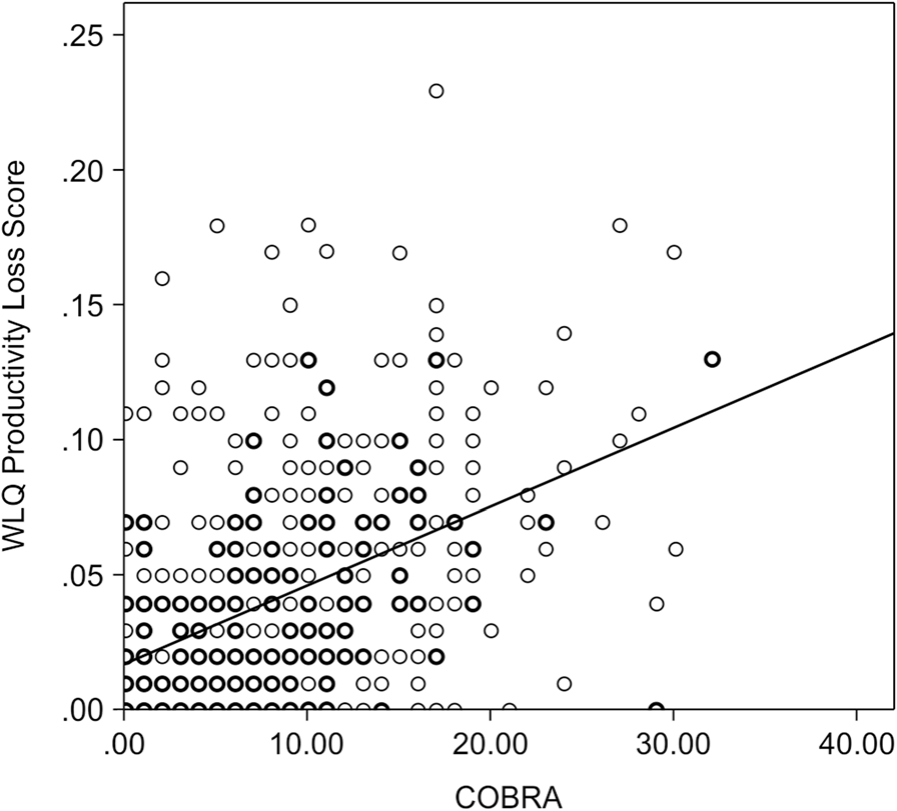
Association between WLQ-8 Productivity loss and COBRA scores (*N =* 476). Formula of regression line: y = 0.003x + 0.018 (y = WLQ-8 Productivity loss, x = COBRA)

Spearman’s correlation analyses confirmed significant associations between subjective cognitive function and the WLQ-8 productivity loss score (ρ = .470, *p* < .01) (Table 2, Fig. 1). The Mann-Whitney U test was conducted to assess the difference in presenteeism measures between the low COBRA score group (COBRA ≤14) and the high COBRA score group (COBRA > 14). The high score group was significantly worse than the low score group in the WLQ-8 productivity loss score (*Z* = − 7.88, *p* < .001).

### Subjective cognitive function, depressive symptoms, and work limitations

**Fig. 2:**
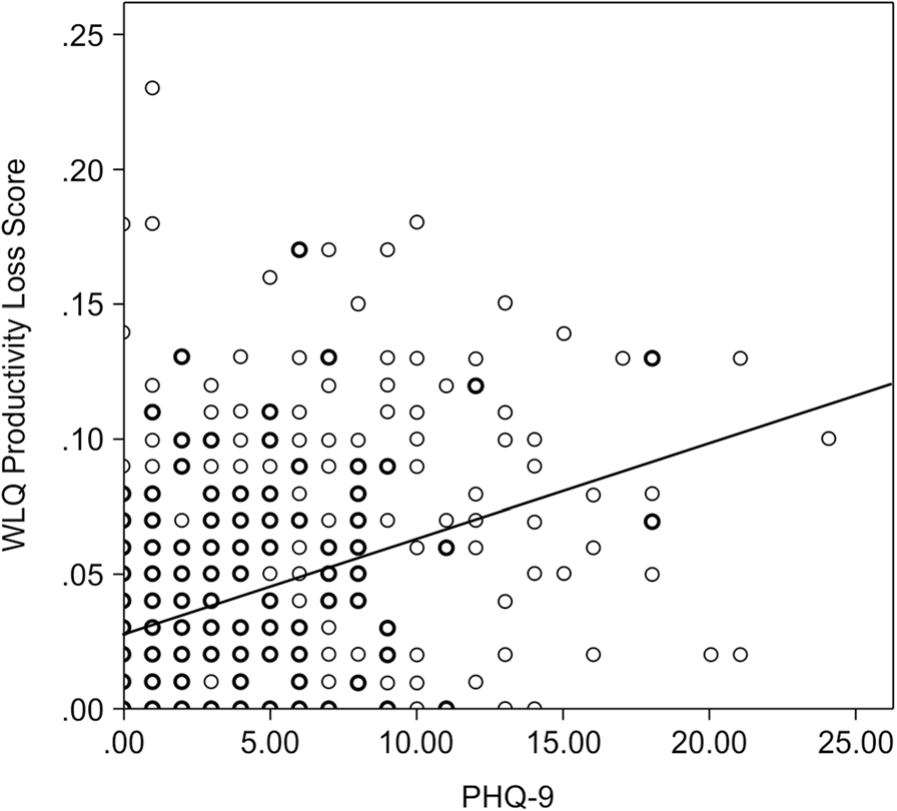
Association between WLQ-8 Productivity loss and PHQ-9 scores (*N =* 476). Formula of regression line: y = 0.004x + 0.027 (y = WLQ-8 Productivity loss, x = PHQ-9)

Spearman’s correlation analyses confirmed a significant association between subjective cognitive function and depression (ρ = .407, *p* < .01; Table 2). The WLQ productivity loss score (ρ = .399, *p* < .01) was significantly associated with the PHQ-9 score (ρ = .399, *p* < .01) (Table 2, Fig. 2).

### Multiple regression analysis of WLQ-8 productivity loss

A hierarchical multiple regression analysis was performed with the WLQ-8 productivity loss score as the dependent variable and COBRA and PHQ-9 as independent variables (predictors; Table 3). The adjusted *R*^2^ was .24 (*p* < .001); cognitive complaints (*β* = 0.36, *p* < .001) and depression (*β* = 0.22, *p* < .001) were significant predictors. No significant interaction was observed between subjective cognitive function and depression (*β* = − 0.015, *p* = .74; ⊿*R*^*2*^ = 0.00, *p* > .05).

**Table 3:** Results of the hierarchical multiple regression analysis of WLQ-8 productivity loss (N = 476)

			Step 1	(F = 74.87, *p* = 0.000)				Step 2	(F = 49.86, *p* = 0.000)	
Variable	*B* (95%CI)	*b* SE	t	*β*	*p*	VIF	t	*β*	*p*	VIF
Step 1										
PHQ-9	0.002 (0.001–0.003)	0.000	4.90	0.22	< 0.001**	1.20				
COBRA	0.002 (0.002–0.003)	0.000	8.22	0.36	< 0.001**	1.20				
Step 2										
PHQ-9 × COBRA							−0.33	−0.015	0.74	1.26
⊿*R*^*2*^			0.24**					0.000		
Adjusted *R*^*2*^			0.24					0.24		

*Abbreviations*: *COBRA* Cognitive Complaints in Bipolar Disorder Rating Assessment; *PHQ-9* Patient Health Questionnaire-9; *WLQ-8* Work Limitations Questionnaire; **p < 0.01 (two-sided)

### Path analysis

**Fig. 3:**
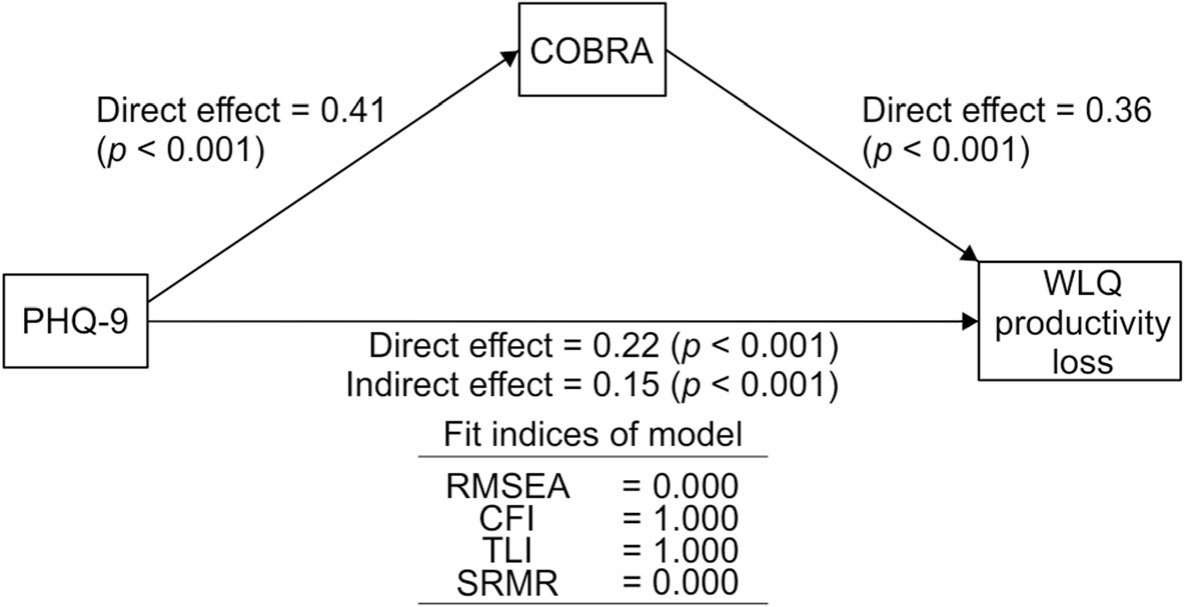
Results of path analysis with PHQ-9, COBRA, and WLQ-8 Productivity loss scores (*N* = 476)

To examine the complex associations between subjective cognitive function, presenteeism, and depressive symptoms, we conducted a path analysis on the outcome of the aforementioned univariate and multiple regression analyses. The standardized path coefficients were computed using depressive symptoms, subjective cognitive function, and WLQ-8 productivity loss. The model was saturated and is shown in Fig. 3. According to this model, subjective cognitive function affected presenteeism directly (direct effect = .36, *p* < .001) and depression affected presenteeism not only directly (direct effect = .22, *p* < .001), but also through cognitive complaints (indirect effect = .15, *p* < .001). Therefore, subjective cognitive function significantly mediated the effects of depressive symptoms on work limitations. In this path analysis, the squared multiple correlation of WLQ-8 productivity loss was .24.

## Discussion

Our study identified a correlation between subjective neuro-cognition and the presenteeism of adult workers. Spearman’s correlation analyses showed significant associations between subjective cognitive impairment and all the presenteeism scores. In the multiple regression analysis, cognitive complaints were a significant predictor of WLQ-8 productivity loss. These findings supported the hypothesis that subjective neuro-cognition is correlated with the presenteeism of adult workers. Our study is in line with other research showing an association between cognitive impairment and presenteeism [10, 26]. We strongly expected subjective cognitive impairment to be correlated with presenteeism, as subjective cognitive dysfunction can easily affect daily work. This finding suggests that cognitive impairment is an important factor of the presenteeism of workers.

It is important to note the necessity of assessing the cognitive function of workers. In this study, we decided to assess the cognitive function of adult workers using the COBRA. Recently, it has been recommended that the assessment of the subjective cognitive function of bipolar patients be done with the COBRA [14]. However, evaluating cognitive function might also be useful for other psychiatric patients and even healthy individuals, in addition to bipolar patients. In Japan, the COBRA has been evaluated for the general population and has been confirmed as a useful tool to assess the subjective cognitive function of adults [18]. The COBRA is a self-evaluation criterion scale; thus, when the respondent has depressive symptoms, cognitive impairment may be evaluated as being worse because of negative thinking. If possible, it is also desirable to conduct objective cognitive function tests to assess cognitive function accurately. However, our study showed that it is meaningful to evaluate cognitive function using the COBRA, even for adult workers.

Regarding the relation between cognitive complaints and presenteeism, our data showed that depressive symptoms were significantly correlated with cognitive complaints and all WLQ-8 scores (Table 2). From the view of presenteeism, according to the multiple regression analysis that used WLQ-8 productivity loss as the outcome, subjective cognitive function and depressive symptoms predict the presenteeism of Japanese adult workers. In a previous study, manager support was related to workplace productivity for employees with depression [27]; thus, in future research, it may be necessary to evaluate the effect of manager support on workers’ subjective cognitive function.

Our study showed that subjective cognitive impairment and presenteeism are closely related, while the indirect effect of depressive symptoms on presenteeism was also significant (Fig. 3). In remitted major depressive disorder, residual depressive symptoms were strongly associated with quality of life, verbal memory was correlated with part of the workers’ quality of life, and these associations may be independent of clinical factors [28]. In the general Japanese adult population, higher WLQ subscale scores were related to depression [29]. Considering these previous studies and our results, cognitive impairment may independently affect quality of life even in the general working adult population; thus, when managing presenteeism, it may be important to consider interventions to cope with cognitive impairment in addition to depressive symptoms. As far as we know, this is the first demonstration of the correlation between cognitive complaints and presenteeism in the general Japanese adult population. This study, therefore, provides a first step toward dealing with presenteeism in Japan.

### Limitations

The cross-sectional study design could be considered a limitation of this study. As this was exploratory research, a correction for multiple comparisons was not applied. Moreover, causal linkages among the parameters could not be established. Participants in this study were adult workers; thus, our results may not be applicable to patients with mental disorders such as bipolar disorder. While the WLQ-8 is a useful and short tool for estimating the loss of productivity (presenteeism), it is a self-report measure that does not capture the actual work performance of an individual.

## Conclusions

Subjective cognitive function, rather than depressive symptoms, may be strongly related to the presenteeism of the adult workers in this community sample. This is the first research study, to our knowledge, that evaluates the correlation between cognitive complaints and presenteeism. Future research needs to investigate the factors of the subjective cognitive impairment of adult workers from the community.

## Data Availability

The datasets of this research are available on request to the corresponding author.

## Abbreviations

CFI: Comparative fit index
CI: Confidence interval
COBRA: Complaints in Bipolar Disorder Rating Assessment
PHQ-9: Patient Health Questionnaire 9
RMSEA: Root mean square error of approximation
SRMR: Standardized root mean squared residual
TLI: Tucker-Lewis index
WLQ-8: Work Limitations Questionnaire 8
